# Ultra-High-Throughput and Low-Volume Analysis of Intact Proteins with LAP-MALDI MS

**DOI:** 10.1021/jasms.3c00068

**Published:** 2023-04-27

**Authors:** Bob Challen, Michael Morris, Rainer Cramer

**Affiliations:** † Department of Chemistry, University of Reading, Whiteknights, Reading RG6 6DX, U.K.; ‡ Waters Corporation, Stamford Avenue, Wilmslow SK9 4AX, U.K.

## Abstract

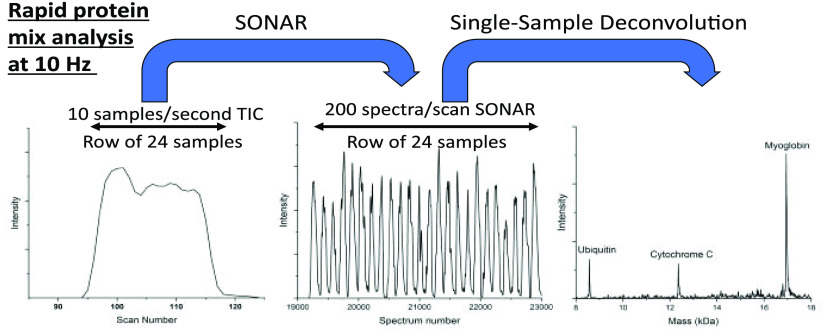

High-throughput (HTP) mass spectrometry (MS) is a rapidly growing field, with many techniques evolving to accommodate ever increasing sample analysis rates. Many of these techniques, such as AEMS and IR-MALDESI MS, require volumes of at least 20–50 μL for analysis. Here, liquid atmospheric pressure-matrix-assisted laser desorption/ionization (LAP-MALDI) MS is presented as an alternative for ultra-high-throughput analysis of proteins requiring only femtomole quantities of protein in 0.5 μL droplets. By moving a 384-well microtiter sample plate with a high-speed XY-stage actuator, sample acquisition rates of up to 10 samples per second have been achieved at a data acquisition rate of 200 spectra per scan. It is shown that protein mixture solutions with concentrations of ≤2 μM can be analyzed at this speed, while individual protein solutions can be analyzed at concentrations of ≤0.2 μM. Thus, LAP-MALDI MS provides a promising platform for multiplexed HTP protein analysis.

## Introduction

The growing field of protein therapeutics has caused a rising demand in high-throughput (HTP) analysis techniques to identify post-translational modifications and protein/small molecule interactions.^1,2^ While top-down^3^ and bottom-up^4^ MS provide high specificity^5^ toward locating these interactions, observing changes to intact proteins from large libraries by high-throughput screening (HTS) could greatly speed up the identification of potential therapeutic proteins.

HTP mass spectrometry (MS) techniques for the analysis of small and 'mid'-sized molecules (e.g., peptides, pharmaceuticals) have reached extreme speeds in recent years, with up to 60 samples/second being demonstrated using liquid atmospheric pressure matrix-assisted desorption/ionization (LAP-MALDI) MS.^6^ Although previously shown to be capable of efficient native protein analysis,^7^ LAP-MALDI MS has yet to be applied in HTP analysis of intact proteins. While conventional solid UV-MALDI MS on axial TOF instrumentation is a more commonly used technique for HTP protein analysis,^8^ the ESI-like multiply charged protein ion species produced by LAP-MALDI MS offer a distinct advantage in terms of mass resolution when using high-performing, hybrid mass spectrometers such as Q-TOF and Orbitrap instrumentation.

Techniques such as acoustic ejection MS (AEMS) and infrared matrix-assisted laser desorption/electrospray ionization (IR-MALDESI) have found recent success in their applications toward the HTP analysis of intact proteins, with sample acquisition rates of 1^9^ and 1.5–22 Hz,^10,11^ respectively. While impressive, both techniques require for the analysis relatively large sample volumes of 20–50 μL. Furthermore, analysis at 22 Hz with IR-MALDESI produced coefficient of variation (CV) values of up to 42%.

With LAP-MALDI MS, extremely low volumes of <1 μL can be analyzed. Sample consumption is also negligible, with previous studies estimating the analyte consumption per laser shot at <30 amol.^12^ Earlier liquid MALDI MS studies demonstrated the use of nL droplets for successful peptide analysis at the femtomole level with a conservative estimate of <1 pL sample consumption per laser shot.^13^ Here, we present LAP-MALDI MS as a platform for HTP intact protein analysis, aiming to push the boundaries of sample acquisition rates while minimizing sample volumes and analyte quantities.

## Experimental Section

### Materials

Myoglobin from equine heart, cytochrome C from bovine heart, ubiquitin from bovine erythrocytes, glycerol, acetonitrile (ACN), water, and α-cyano-4-hydroxycinnamic acid (CHCA) were all purchased from Sigma-Aldrich (Gillingham, UK).

### Matrix, Analyte, and Sample Preparation

For the liquid support matrix (LSM), a 5 mg/mL solution of CHCA was prepared in 1 volume of H_2_O/ACN (3:7), to which 0.6 volumes of glycerol were added. Myoglobin, cytochrome C, and ubiquitin were dissolved in water to stock concentrations of 100 μM. For the preparation of the mixed protein solution, the three proteins were mixed in a 1:1:1 ratio. All protein solutions were then diluted to a range of 0.33–33 μM and mixed 1:1 with the LSM for a final concentration range of 0.17–17 μM. When spotting the combined matrix/analyte solutions onto the sample plates, volumes of 2 μL were used for the 96-well quarter-size microtiter plate format, and volumes of 0.5 μL were used for the 384-well full-size microtiter plate format.

### LAP-MALDI MS Setup and Analysis

All experiments utilized a Synapt G2-Si (Waters, Wilmslow, UK) fitted with a custom LAP-MALDI ion source, described in detail elsewhere.^14^ Briefly, a steel LAP-MALDI sample plate (either 96- or 384-well) was positioned orthogonally at a 3 mm distance to a heated inlet ion transfer tube with a nitrogen counter-gas flow of 220 L/h. The beam of a 2 kHz pulsed 343 nm diode-pumped solid-state laser (FlareNX 343–0.2–2; Coherent, Santa Clara, USA) was aligned at a 60° angle to the sample plate, providing a laser energy of approximately 10 μJ/pulse on the sample droplets. A high-speed XY-stage was used to move the target plate, controlled via a Python script. The instrument acquisition mode was set to SONAR,^15^ with the quadrupole set to RF-only mode and with disabled scanning to minimize the interscan-delay time. The ion mobility gases were also disabled.

All samples were analyzed by rastering the stage in a serpentine motion at speeds of 2.5–25 mm/s. All mass spectra were processed within MassLynx (Waters), and all deconvolutions were performed with UniDec.^16^

## Results and Discussion

Initially, 2-μL droplets of a mixed protein solution were spotted on a 96-well sample plate and run without using SONAR at an analysis speed of 2 samples/s. Although already rapid, any greater analysis speed (by increasing the translational speed of the XY-stage) caused undersampling with only three or less data points per sample. The instrument’s data acquisition scan rate is limited to 10 Hz and results in reduced ion signal detection at high scan rates due to the lack of ion signal recording during the interscan-delay time, which becomes relatively large compared to the time of ion signal detection at higher scan rates.

**Figure 1:**
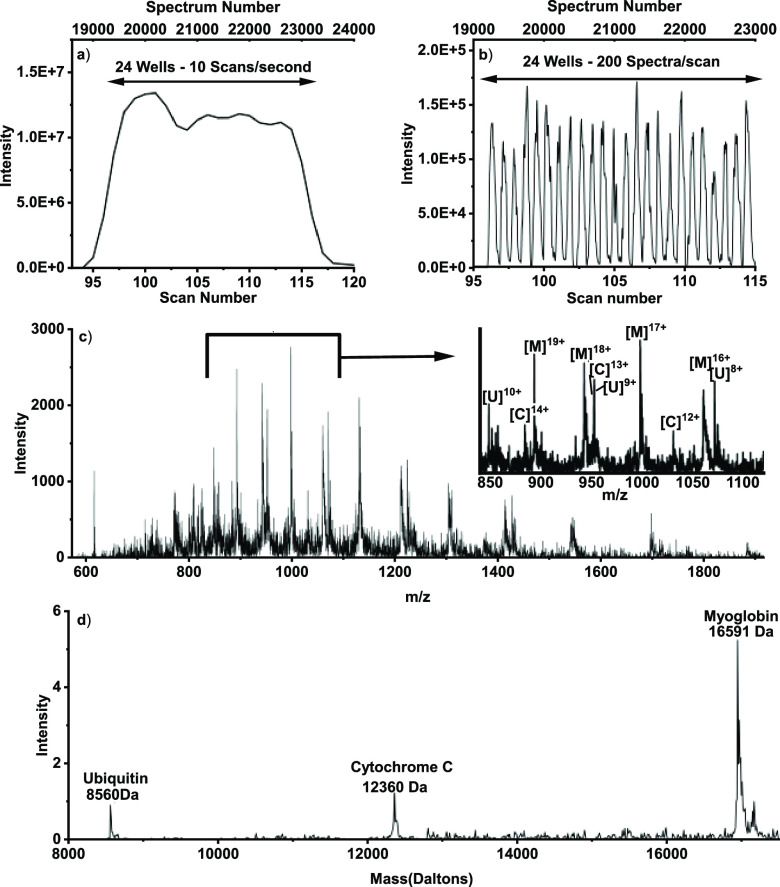
HTP LAP-MALDI MS analysis of a protein mixture using SONAR data acquisition. A 24-well analysis of a 17 μM mixture of proteins using a sample volume of 0.5 μL produces the TIC in (a). Depicting the total ion current per individual spectrum for the 200 binned spectra/scan results in well-resolved peaks for each sample as seen in (b). Panel (c) shows the combined ion signal of all mass spectra from a single peak in (b) with an inlaid zoomed-in section for *m*/*z* 850–1100, where *U*, *C*, and *M* represent ubiquitin, cytochrome C and myoglobin, respectively. Panel (d) shows the result of the deconvolution of the mass spectrum in (c).

To overcome this limitation, the SONAR acquisition mode was used. In brief, SONAR allows for each TOF scan to bin 200 spectra, greatly increasing the temporal resolution of the data acquisition as demonstrated in Figure 1a,b. Figure 1a shows the scan-based total ion chromatogram (TIC) for a single 24-well row of a 384-well microtiter plate at a speed of 10 samples/s. As can be seen, the temporal resolution using the accumulated scan data is not high enough to distinguish each individual sample.

By using SONAR, however, the data of each scan can be recorded within 200 individually binned spectra per scan, allowing for lower scan rates but overall higher data acquisition rates, thus avoiding undersampling. The loss of ion signal due to the interscan-delay times is also substantially reduced, as these only occur after each scan, i.e., after 200 spectra. In Figure 1b, the TIC scan data from Figure 1a are shown by depicting the total ion current per individual spectrum, revealing the greatly increased temporal resolution gained by acquiring the data in SONAR mode. Each resolved peak has sufficient data points and provides sufficient ion signal intensity to observe the expected profiles of multiply charged proteins (Figure 1c), resulting in high-quality deconvoluted LAP-MALDI protein mass spectra (Figure 1d). At all acquisition speeds (2–10 samples/second), a mass spectral resolution of 8000–10 000 is easily achievable for all proteins.

**Figure 2:**
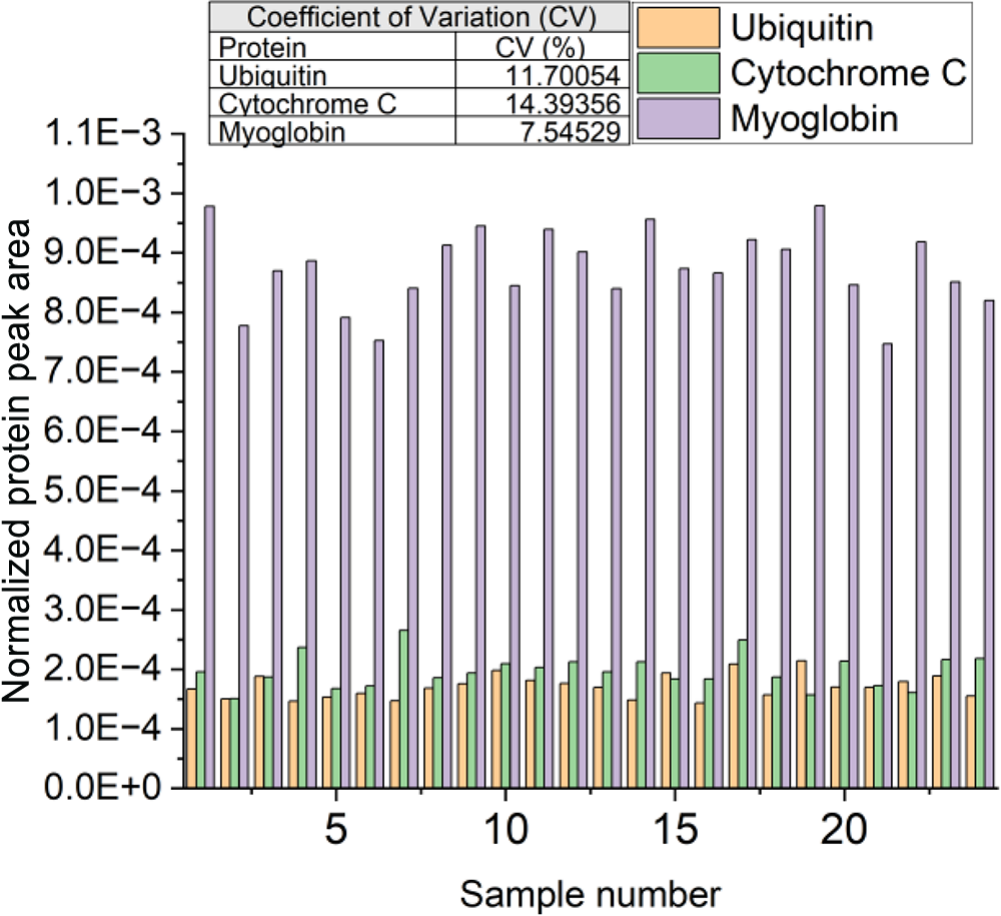
Total ion current-normalized LAP-MALDI MS peak areas for a 17 μM protein mixture of ubiquitin, cytochrome C, and myoglobin. The inset table shows the calculated CVs for each protein across the whole data set.

While high analysis speeds are desirable, if the droplet-to-droplet ion signal stability is poor, the data are generally rendered meaningless. The CV for each row of samples was therefore calculated by normalizing the deconvoluted protein peak area to the total ion current of each sample. As seen in Figure 2, the CV for each deconvoluted protein peak area was <15%. The CV would likely be improved with liquid handling robots, as the precision (and accuracy) of the dispensed volumes are often much better than those obtained by manual sample spotting. Spotting volume consistency is vital as the volume greatly affects the shape of the droplet. As the distance of the heated capillary to the sample affects signal intensity,^17^ it can be expected that even small variations in droplet size will consequently affect the signal intensity.

**Figure 3:**
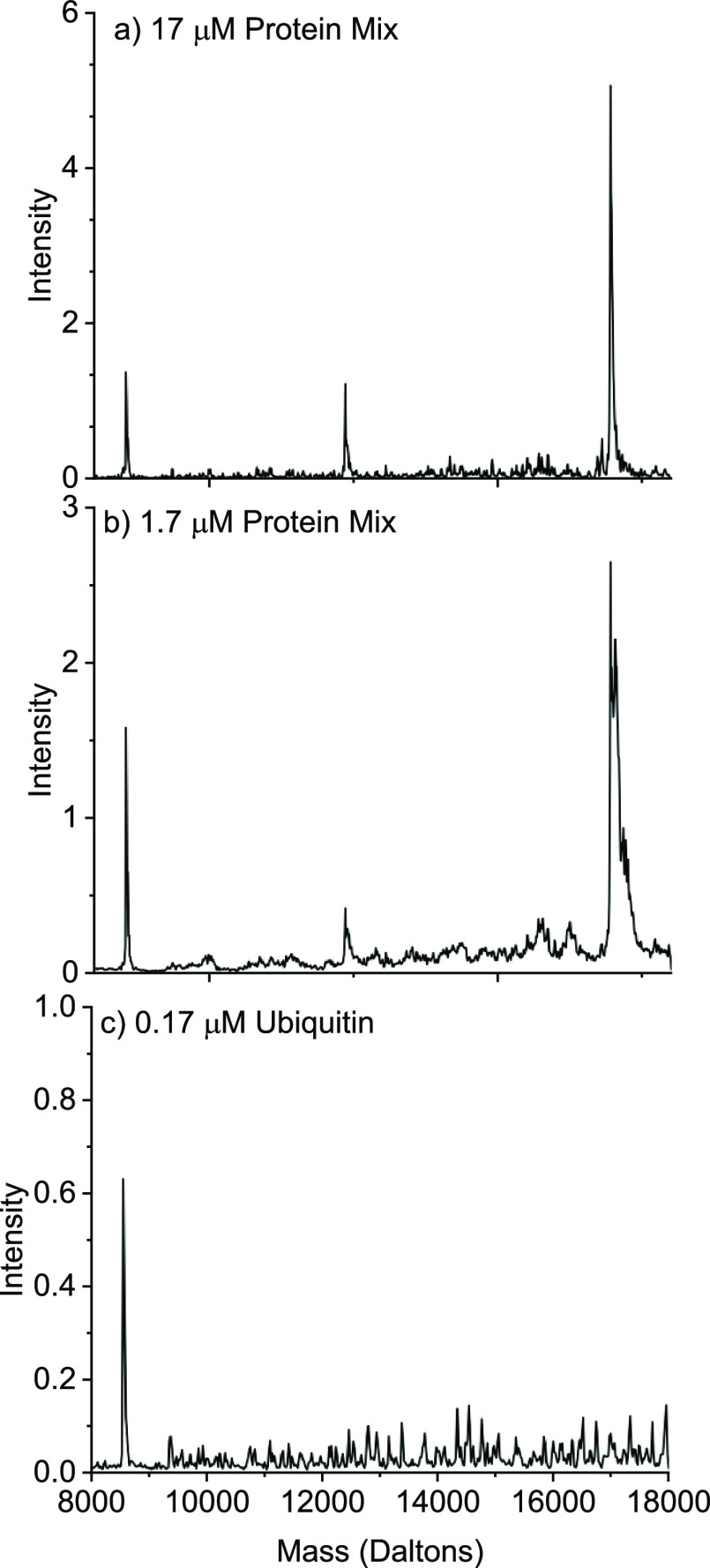
Deconvoluted LAP-MALDI mass spectra for (a) a 17 μM protein mixture, (b) a 1.7 μM protein mixture, and (c) a 0.17 μM ubiquitin-only sample. All spectra are the results of combining the data from one sample droplet (approximately 140 spectra/sample droplet) at a sample analysis speed of 10 samples/s using a data acquisition speed of 10 scans/s.

An additional benefit of LAP-MALDI is the low sample volume required—just ≤0.5 μL is needed per sample droplet. Figure 3a,b displays the deconvoluted mass spectra from a single 0.5 μL droplet of the protein mixture (analyzed at 10 samples/s and 10 scans/s) at concentrations of 17 and 1.7 μM per protein, respectively. While 1.7 μM is a comparable concentration to limits reached by other HTP protein MS analysis approaches,^8,9^ the absolute amount of protein used per sample droplet is around 40 times smaller than shown by other techniques, with only 850 fmol of protein needed.

At concentrations of 0.17 μM per protein and below, ion suppression effects in the protein mixture samples possibly increased to the point where the suppressed proteins were unable to be detected. When analyzing samples with a single protein analyte, it is still possible to detect the individual protein and to deconvolute the obtained protein mass spectrum when only 85 fmol of protein is present in the sample droplet (Figure 3c).

## Conclusions

In this work, the use of LAP-MALDI MS in HTP analysis of proteins and protein mixtures has been demonstrated. Speeds of up to 10 samples per second have been easily achieved, with the potential for higher speeds. This level of sample analysis speed and throughput exceeds current HTP methods for intact protein analysis by 10-fold while reducing absolute sample amounts 40-fold. Even without using SONAR, LAP-MALDI MS can still be used to analyze intact proteins at a rate of 2 samples/s, showing that LAP-MALDI MS is a powerful technique for improving sample throughput employing current instrumentation.

## Data Availability

Data supporting the results reported in this paper are openly available from the University of Reading Research Data Archive at 10.17864/1947.000456.

## References

[ref1] Leader B.; Baca Q. J.; Golan D. E. Protein therapeutics: a summary and pharmacological classification. Nat. Rev. Drug Discovery 2008, 7 (1), 21–39. 10.1038/nrd2399. 18097458

[ref2] Chen G.; Warrack B. M.; Goodenough A. K.; Wei H.; Wang-Iverson D. B.; Tymiak A. A. Characterization of protein therapeutics by mass spectrometry: recent developments and future directions. Drug Discov 2011, 16 (1), 58–64. 10.1016/j.drudis.2010.11.003. 21093608

[ref3] Catherman A. D.; Skinner O. S.; Kelleher N. L. Top Down proteomics: Facts and perspectives. Biochem. Biophys. Res. Commun. 2014, 445 (4), 683–693. 10.1016/j.bbrc.2014.02.041. 24556311 PMC4103433

[ref4] Rogawski R.; Sharon M. Characterizing Endogenous Protein Complexes with Biological Mass Spectrometry. Chem. Rev. 2022, 122 (8), 7386–7414. 10.1021/acs.chemrev.1c00217. 34406752 PMC9052418

[ref5] Aebersold R.; Mann M. Mass spectrometry-based proteomics. Nature 2003, 422 (6928), 198–207. 10.1038/nature01511. 12634793

[ref6] Krenkel H.; Brown J.; Richardson K.; Hoyes E.; Morris M.; Cramer R. Ultrahigh-Throughput Sample Analysis Using Liquid Atmospheric Pressure Matrix-Assisted Laser Desorption/Ionization Mass Spectrometry. Anal. Chem. 2022, 94 (10), 4141–4145. 10.1021/acs.analchem.1c05614. 35234449 PMC9385107

[ref7] Hale O. J.; Cramer R. Atmospheric Pressure Ultraviolet Laser Desorption and Ionization from Liquid Samples for Native Mass Spectrometry. Anal. Chem. 2019, 91 (22), 14192–14197. 10.1021/acs.analchem.9b03875. 31651149 PMC7007007

[ref8] Dueñas M. E.; Peltier-Heap R. E.; Leveridge M.; Annan R. S.; Büttner F. H.; Trost M. Advances in high-throughput mass spectrometry in drug discovery. EMBO Mol. Med. 2023, 15 (1), e14850 10.15252/emmm.202114850. 36515561 PMC9832828

[ref9] Zacharias A. O.; Liu C.; VanAernum Z. L.; Covey T. R.; Bateman K. P.; Wen X.; McLaren D. G. Ultrahigh-Throughput Intact Protein Analysis with Acoustic Ejection Mass Spectrometry. J. Am. Soc. Mass Spectrom. 2023, 34 (1), 4–9. 10.1021/jasms.2c00276. 36468949

[ref10] Pu F.; Ugrin S. A.; Radosevich A. J.; Chang-Yen D.; Sawicki J. W.; Talaty N. N.; Elsen N. L.; Williams J. D. High-Throughput Intact Protein Analysis for Drug Discovery Using Infrared Matrix-Assisted Laser Desorption Electrospray Ionization Mass Spectrometry. Anal. Chem. 2022, 94 (39), 13566–13574. 10.1021/acs.analchem.2c03211. 36129783

[ref11] Radosevich A. J.; Pu F.; Chang-Yen D.; Sawicki J. W.; Talaty N. N.; Elsen N. L.; Williams J. D.; Pan J. Y. Ultra-High-Throughput Ambient MS: Direct Analysis at 22 Samples per Second by Infrared Matrix-Assisted Laser Desorption Electrospray Ionization Mass Spectrometry. Anal. Chem. 2022, 94 (12), 4913–4918. 10.1021/acs.analchem.1c04605. 35290016

[ref12] Cramer R.; Pirkl A.; Hillenkamp F.; Dreisewerd K. Liquid AP-UV-MALDI Enables Stable Ion Yields of Multiply Charged Peptide and Protein Ions for Sensitive Analysis by Mass Spectrometry. Angew. Chem., Int. Ed. 2013, 52 (8), 2364–2367. 10.1002/anie.201208628. PMC3592991 23341077

[ref13] Palmblad M.; Cramer R. Liquid matrix deposition on conductive hydrophobic surfaces for tuning and quantitation in UV-MALDI mass spectrometry. J. Am. Soc. Mass Spectrom. 2007, 18 (4), 693–697. 10.1016/j.jasms.2006.11.013. 17223354

[ref14] Krenkel H.; Hartmane E.; Piras C.; Brown J.; Morris M.; Cramer R. Advancing Liquid Atmospheric Pressure Matrix-Assisted Laser Desorption/Ionization Mass Spectrometry Toward Ultrahigh-Throughput Analysis. Anal. Chem. 2020, 92 (4), 2931–2936. 10.1021/acs.analchem.9b05202. 31967792 PMC7145281

[ref15] Gethings L. A.; Richardson K.; Wildgoose J.; Lennon S.; Jarvis S.; Bevan C. L.; Vissers J. P. C.; Langridge J. I. Lipid profiling of complex biological mixtures by liquid chromatography/mass spectrometry using a novel scanning quadrupole data-independent acquisition strategy. Rapid Commun. Mass Spectrom. 2017, 31 (19), 1599–1606. 10.1002/rcm.7941. 28703389

[ref16] Marty M. T.; Baldwin A. J.; Marklund E. G.; Hochberg G. K. A.; Benesch J. L. P.; Robinson C. V. Bayesian Deconvolution of Mass and Ion Mobility Spectra: From Binary Interactions to Polydisperse Ensembles. Anal. Chem. 2015, 87 (8), 4370–4376. 10.1021/acs.analchem.5b00140. 25799115 PMC4594776

[ref17] Ryumin P.; Brown J.; Morris M.; Cramer R. Investigation and optimization of parameters affecting the multiply charged ion yield in AP-MALDI MS. Methods 2016, 104, 11–20. 10.1016/j.ymeth.2016.01.015. 26827934

